# Human Tularemia Epididymo-Orchitis Caused by *Francisella tularensis* Subspecies *holartica*, Austria

**DOI:** 10.3201/eid2910.230436

**Published:** 2023-10

**Authors:** Maximilian Seles, Julia Altziebler, Gregor Gorkiewicz, Lisa Kriegl, Stefan Hatzl, Sascha Ahyai, Romana Klasinc, Ines Zollner-Schwetz, Robert Krause

**Affiliations:** Medical University of Graz, Graz, Austria (M. Seles, J. Altziebler, G. Gorkiewicz, L. Kriegl, S. Hatzl, S. Ahyai, I. Zollner-Schwetz, R. Krause);; Medical University of Vienna, Vienna, Austria (R. Klasinc)

**Keywords:** tularemia, Francisella tularensis subspecies holartica, bacteria, epididymo-orchitis, orchiectomy, human tularemia, zoonoses, Austria

## Abstract

A previously healthy man in Austria had tularemia epididymo-orchitis develop, leading to unilateral orchiectomy. *Francisella tularensis* subspecies *holartica* was detected by 16S rRNA gene sequencing analysis of inflamed granulomatous testicular tissue. Clinicians should suspect *F. tularensis* as a rare etiologic microorganism in epididymo-orchitis patients with relevant risk factors.

Tularemia is a highly pathogenic zoonosis caused by the gram-negative intracellular bacterium *Francisella tularensis* (*1*). *F. tularensis* subspecies *tularensis* (type A) and *holarctica* (type B) are the main causative agents for tularemia in humans and animals; type B is present in Europe and Asia (*2*). Humans are infected through bites of arthropods (including ticks that are the primary vector of tularemia), as well as by inhaling infectious aerosols, handling infected animals, or ingesting contaminated water or food (*3*). Tularemia cases caused by inadvertent exposure among laboratory personnel have also been reported (*4*).

Clinical manifestations of tularemia in humans might result in ulceroglandular, oculoglandular, glandular, oropharyngeal, pulmonary, or typhoidal forms of disease. We report a case of tularemia epididymo-orchitis in a healthy man that led to unilateral orchiectomy.

## The Study

In July 2022, a previously healthy 69-year-old man (nature filmmaker) came to an outpatient clinic in Austria because of fever (temperature up to 39°C), chills, malaise, headache, and lower abdominal pain after traveling to southern Slovenia, Cres island (Croatia), and northern Styria (Austria) 1 month earlier. The patient reported several tick bites but no further animal contact. Clinical examination showed a small ulcerative lesion on the lower left back, which was initially suspected to be an infected insect bite.

Laboratory tests showed leukocytosis (13.6 × 10^9^ cells/L), increased C-reactive protein (CRP) of 85 mg/L, a serum creatinine level of 1.17 mg/mL, and an estimated low glomerular filtration rate of 63 mL/min/1.73m^2^. The patient was admitted and initially given amoxicillin/clavulanic acid, which was subsequently changed to piperacillin/tazobactam plus moxifloxacin 3 days later because of persistent fever and sudden testicular swelling and pain, as well as an increased CRP level (357 mg/L) and leukocyte count (22.5 × 10^9^ cells/L).

**Figure 1:**
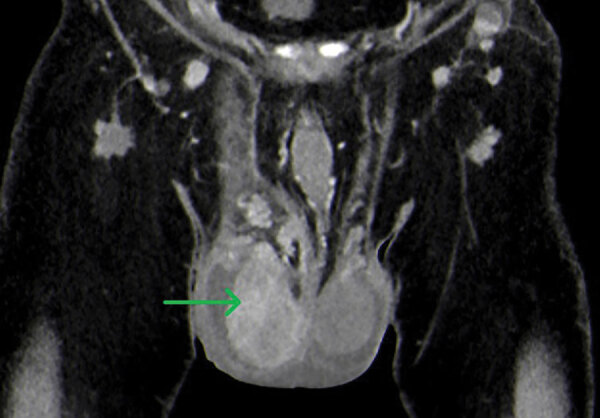
Computed tomography of patient who had human tularemia epididymo-orchitis caused by *Francisella tularensis* subspecies *holartica*, Austria. Coronal image shows the right testicle (arrow) during the arterial phase with hyperperfusion and nonperfused areas (abscess).

Computed tomography of the thorax, abdomen, and pelvis showed bilateral epididymo-orchitis and an enlarged right testicle with hyperperfusion and nonperfused areas and a hypoperfused left testicle (Figure 1). In addition, a pulmonary infiltration (diameter 5 mm) in the left lower lobe and diverticulitis were detected. Results of blood and urine cultures were negative. Urine antigen test results for *Legionella* sp. and pneumococci showed negative results. Antibodies specific for *Brucella* spp., *Leptospira* spp., and HIV were not detected.

The patient was transferred to the Medical University of Graz, where piperacillin/tazobactam was continued and moxifloxacin stopped. During the next few days, sonographic examinations showed an enlarged and inhomogenous and hyperperfused right testicle with clinical epididymitis but decreasing CRP levels. Persistent pain and progressive inflammation observed by testicular ultrasound, including suspected abscess, led to unilateral orchiectomy (right testicle) 14 days after admission. Intraoperatively, the testicle and the spermatic cord showed massive inflammation. The orchiectomy was performed without any complications.

**Figure 2:**
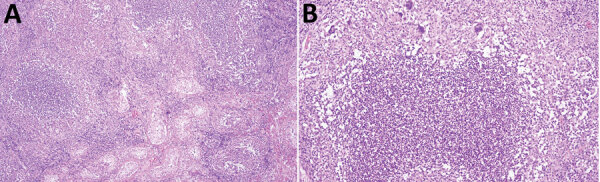
Granulomatous epididymo-orchitis in patient who had human tularemia epididymo-orchitis caused by *Francisella tularensis* subspecies *holartica*, Austria. A) Diffuse chronic granulomatous inflammation in the interstitium and between the seminiferous tubule. Hematoxylin and eosin stain; original magnification ×20. B) Suppurative granuloma with epithelioid cells and single giant cells in testicular tissue. Hematoxylin and eosin stain; original magnification ×100.

The leukocyte count returned to the reference range, and the CRP level decreased to 68 mg/L after surgery. Histopathologic examination showed a chronic granulomatous epididymo-orchitis with abundant suppurative granulomas located between destroyed seminiferous tubules in addition to diffuse mixed interstitial inflammatory infiltrate (Figure 2). PCRs results for *Mycobacterium tuberculosis* complex and atypical mycobacteria were negative. A 16S rRNA gene sequencing analysis of testicular tissue using an IonTorrent Platform (https://www.thermofisher.com) showed abundant bacterial DNA with 100% homology for *F. tularensis* subspecies *holartica* (78% of generated reads) (*1*).

Subsequently, a commercially available ELISA (Virion/Serion, https://www.virion-serion.de) detected IgM and IgG for a panel of human pathogens in serum or plasma (cutoff value >15 units/mL for IgG and IgM) and indicated the presence of *F. tularensis* lipopolysaccharide. *F. tularensis* antibody levels were 136 units/mL for IgG and >400 units/mL for IgM. The patient received doxycycline plus moxifloxacin for 2 months. At a 6-month follow-up, the patient had no additional complaints.

## Conclusions

In Austria, antibodies against *F. tularensis* are found in 0.5% of healthy adults (*5*), and annual cases range between 0 and 58 (*2**,**6*). In clinically apparent infections, the most frequent manifestations of human tularemia are ulceroglandular or glandular forms. The oculoglandular, oropharyngeal, or pulmonary forms have been less frequently reported (*2*). In the case we report, the patient did not report any direct animal contact or use of unprocessed water or food during his nature filming activities. However, initial examination showed a small ulcerative lesion on the lower left back, which presumably was the initial tularemia skin lesion.

Worldwide, tularemia orchitis has been reported in hares (including 1 case with epididymo-orchitis) (*7**,**8*), a squirrel (*9*), and a marmoset (*10*). Infectious human epididymo-orchitis is usually caused by *Neisseria gonorrhoeae*, *Chlamydia trachomatis*, *Ureaplasma* spp., *Mycoplasma genitalium*, *Escherichia coli*, *Pseudomonas aeruginosa*, and other gram-negative bacteria, as well as *Staphylococcus aureus* in elderly persons. Granulomatous epididymo-orchitis is rare and usually caused by *Mycobacterium tuberculosis* or *Brucella* spp. Other rare etiologic agents include fungi, *Shistosoma* spp., or *Orientia tsutsugamushi* (*11**–**13*). *F. tularensis* has not been previously reported as a causative microorganism for epididymo-orchitis.

Patients who have epididymo-orchitis typically have acute onset unilateral scrotal pain, swelling, and erythema, and treatment with ceftriaxone combined with doxycycline or levofloxacin is recommended (*13*). Tularemia is treated with fluorochinolones, doxycycline, or aminoglycosides depending on disease severity. Our patient empirically received piperacillin/tazobactam with moxifloxacin, but moxifloxacin was discontinued after 3 days. During piperacillin/tazobactam monotherapy, CRP levels decreased, but sonography and clinical status worsened, leading to unilateral orchiectomy. We assume that the empirical application of moxifloxacin for 3 days lowered systemic inflammatory parameters but was too short for improvement of the testicular infection. Ultimately, the etiology of epididymo-orchitis could be elucidated by 16S rRNA gene sequencing analysis, which in this case led to successful directed therapy with doxycycline and moxifloxacin. Because of impaired renal function, aminoglycosides were not considered. Furthermore, serologic analysis confirmed this unusual case of tularemia.

The specific source of tularemia in this case remains unknown because *F. tularensis* subsp. *holartica* was prevalent in all countries visited by the patient before his infection (tularemia cases are reported from Austria and Slovenia and, rarely, from Croatia) (*14*). Nevertheless, our findings indicates that, in patients suspected of having tularemia by medical history (e.g., arthropod bites, animal contact) or clinical examination (e.g., ulcer, rash, lymphadenopathy), clinicians should consider *F. tularensis* as a rare etiologic microorganism in epididymo-orchitis.
